# Seismic survey noise reduces fin whale vocalisations offshore northwestern Spain

**DOI:** 10.1038/s41598-026-40903-x

**Published:** 2026-02-25

**Authors:** Elodie A. Edwards, Amy M. Feakes, Abdullah A. Olcay, Timothy A. Minshull

**Affiliations:** 1 School of Ocean and Earth Science, University of Southampton, Southampton, United Kingdom; 2 Institute of Sound and Vibration Research, University of Southampton, Southampton, United Kingdom

**Keywords:** Fin whales, Seismic surveys, Anthropogenic noise, Airgun, Deep learning, Ecology, Ecology, Ocean sciences

## Abstract

The ever-increasing level of anthropogenic noise in the ocean is a global concern, yet its impacts on marine life remain poorly understood. Offshore seismic surveys generate intense and repetitive sound pulses during the firing of airguns, which potentially affect the behaviour of marine taxa including marine mammals, but fine-scale behavioural impacts are rarely quantified. We assessed the impact of a 2013 seismic survey offshore northwestern Spain on fin whale vocalisations. Using a convolutional neural network trained on 50 h of manually labelled acoustic data (accuracy 85.6%, AUC 0.93), 63 days of continuous ocean-bottom recordings were analysed across four alternating periods with and without airgun shooting. Across three instruments, vocalisation detections decreased by 70% on average during shooting, with reductions of 52% after correcting for worst-case scenario masking. A negative binomial mixed-effects model indicated statistically significant (p = < 0.0001) reductions in calling during shooting compared to quiet periods. Calling declined rapidly within 1–2 days after shooting began, suggesting reduced vocalisation or temporary displacement. While the broader ecological implications cannot be determined from this dataset, these findings indicate short-term behavioural responses to seismic activity and underscore the need for further research into potential consequences for communication, foraging, and habitat use.

**Supplementary Information:**

The online version contains supplementary material available at 10.1038/s41598-026-40903-x.

## Introduction

The level of anthropogenic noise in the ocean is rising continuously as a consequence of the ongoing expansion and intensification of human activities^1–3^. A systematic review of the relevant research available from 1973 to 2020 regarding the impact of anthropogenic noise on marine animals found that 81–94% of studies reported significant negative effects of anthropogenic noise across a range of marine taxa^4^. Marine mammals are especially vulnerable to these impacts due to their reliance on sound for essential life functions such as communication, navigation, and foraging^5–7^. Documented effects of noise on marine mammals include acoustic masking, avoidance or evasive movement, altered vocalisation patterns and elevated stress levels^5,8,9^.

Among anthropogenic sound sources, seismic surveys are of particular concern. This geophysical exploration technique is widely employed for the purpose of seabed mapping and hydrocarbon exploration, typically carried out by the deployment of vessel-towed airgun arrays to produce high-resolution images of the Earth’s subsurface^10^. The repeated firing of these airguns is commonly referred to as ‘shooting’. During shooting, the rapid expansion and contraction of the air bubble formed during the airguns’ high-pressure release generates sound waves that propagate into the surrounding water column and subsurface beneath^2^. The returning sound signals are detected by receivers in the ocean and processed to image the subsurface structure. Airguns produce intense and impulsive sounds, typically recurring every 10–20 s, with low-frequency peak spectral energy at 5–300 Hz and source levels of 200–250 dB^2,11^. These airgun pulses can propagate over long distances, having been recorded more than 3,000 km from their source^12^, while their dominant frequencies overlap with the vocalisation range of baleen whales (10 Hz to 1 kHz)^13^. Given that sound is fundamental for marine mammal communication and survival, this overlap raises particular concerns about the impacts of seismic airgun activity on these populations^4,14–16^.

Baleen whales are often adversely affected by anthropogenic noise, with 85% of relevant studies reporting negative impacts^4^. In response to seismic survey noise, research to date has reported a variety of behavioural responses in baleen whales. For example, bowhead whales (*Balaena mysticetus*) avoided areas within 20 km of active airgun operations^17^, while gray whales (*Eschrichtius robustus*) altered their movement and respiration in response to increasing sound exposure from seismic survey vessels^18^. Other studies have observed a range of acoustic changes in baleen whales exposed to seismic surveys, including alterations in vocalisation patterns and call rates in bowhead whales (*Balaena mysticetus*)^19^, fin whales (*Balaenoptera physalus)*^14^, blue whales (*Balaenoptera musculus*)^15^ and humpback whales (*Megaptera novaeangliae*)^20,21^. However, some studies have found no significant correlations between seismic activity and whale behaviour^22,23^.

**Fig. 1:**
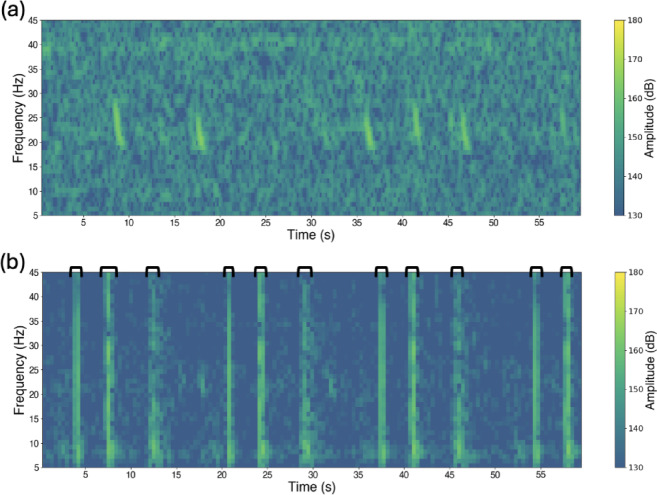
Spectrograms of fin whale pulses recorded by ocean-bottom seismometer (OBS) instruments. **(a)** Example of the low frequency, downsweep structure of the fin whale pulses from Julian Day 175, 2013 (OBS18). **(b)** A segment from Julian Day 198, 2013 (OBS46), recorded during active shooting period, showing fin whale calls at approximately 17 and 32 s occurring between airgun pulses. The black brackets above the plot indicate the portions used to estimate the average masking duration of individual airgun pulses (mean = 5.5 s, range = 2.7–7.7 s; calculated from 20 representative pulses across both survey legs). The spectrograms were generated using a 256-point FFT, 80% overlap, a Hanning window, and a frequency range of 5–45 Hz.

Fin whales are widely distributed throughout the world’s oceans and are classified as Vulnerable by the IUCN Red List, due to ongoing concerns about their conservation status^24^. They use low-frequency vocalisations, the most commonly observed being the ‘20 Hz pulse’^25,26^, that are the focus of this study (Fig. 1). Although the precise function of this call remains uncertain, repetitive and stereotyped sequences of 20 Hz pulses have been suggested to represent reproductive displays^25^ produced by males^27^, whilst isolated or irregular pulses may occur across different demographic groups and appear linked to social or other behavioural contexts^28–30^. The overlap between these vocalisations and the dominant energy of seismic airgun pulses raises concerns of potential acoustic masking, behavioural disruption and/or spatial displacement effects on the species. Despite such concerns, very few studies have examined the responses of fin whales to seismic activity, with the limited evidence indicating changes in vocal behaviour and potential large-scale spatiotemporal avoidance of survey areas^14^.

**Fig. 2:**
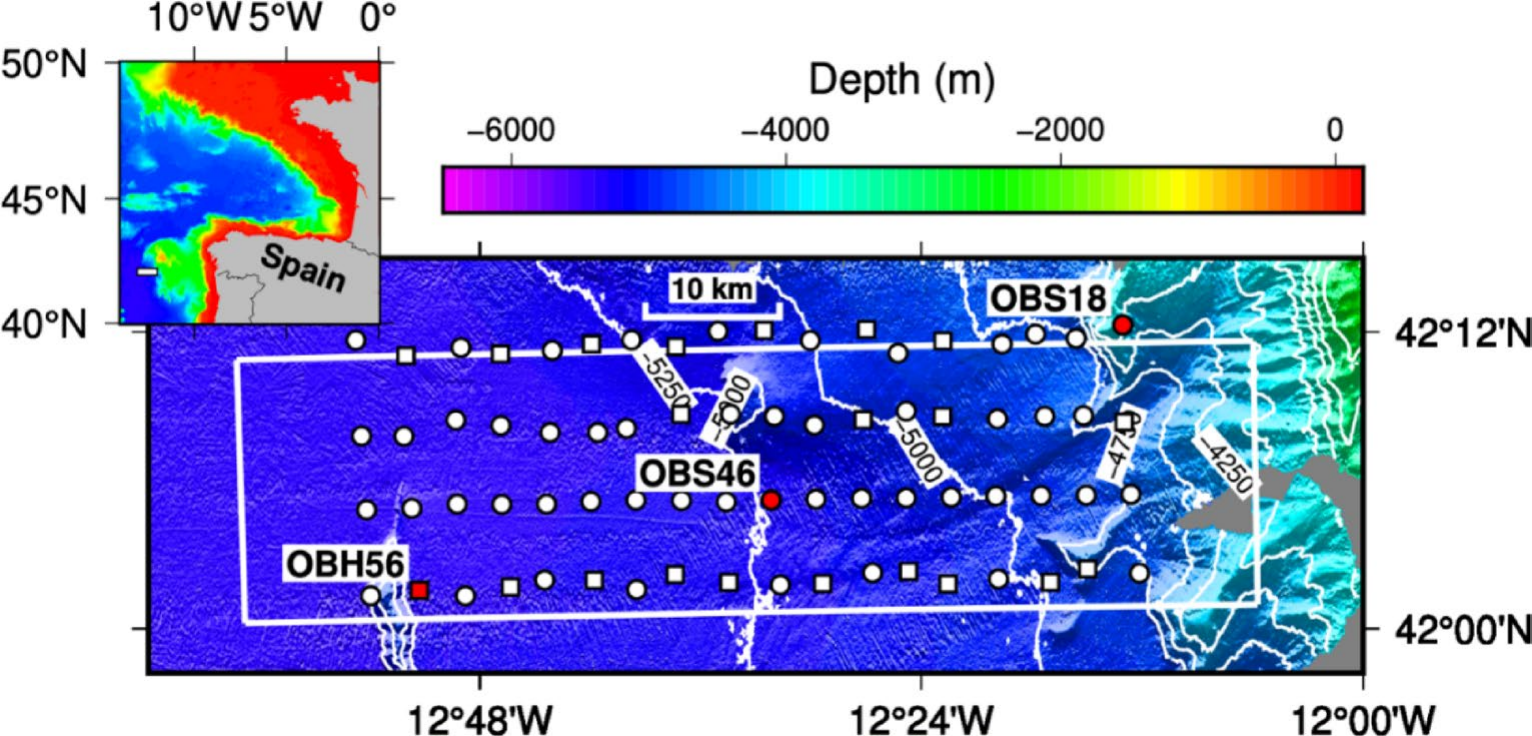
Bathymetry of survey area offshore Galicia Bank, with the grey colour representing areas without swath bathymetric coverage. White symbols mark seafloor instruments deployed, with circles marking ocean-bottom seismometers (OBSs) and squares marking ocean-bottom hydrophones (OBHs). Red symbols represent instruments from which data for this study were obtained. White box marks area of 3D seismic survey. Sail lines were parallel to the long side of the box and at 400 m spacing (see Fig. S2 of Bayrakci et al., 2016). Inset shows regional location of map area offshore Iberia (small white box).

Located offshore northwestern Spain, the Deep Galicia Margin lies along known fin whale migratory routes^31^. In 2013, a large-scale 3D multichannel seismic reflection and wide-angle seismic survey was conducted in this region, including the deployment of 72 ocean-bottom recording instruments across an area of approximately 65 × 25 km (Fig. 2)^32,33^. Two 3,300 cubic inch airgun arrays were fired alternately from *RV Marcus Langseth* at ~ 16-second intervals along 50 parallel survey lines, with a quiet interlude during vessel repairs in Vigo port providing a natural contrast between periods with and without shooting. This study used these contrasting periods to investigate potential impacts of seismic survey activity on fin whale acoustic behaviour. We used a Convolutional Neural Network (CNN) model to detect fin whale 20 Hz pulses within the passive acoustic recordings. By comparing vocal activity across periods of active airgun firing and relative quiet, we evaluated whether seismic operations influenced fin whale calling behaviour. This approach provided novel insights into how high amplitude, temporally persistent, and spatially pervasive anthropogenic noise can alter calling behaviour of fin whales along a key migratory corridor.

## Results

### Machine learning *model *

**Fig. 3:**
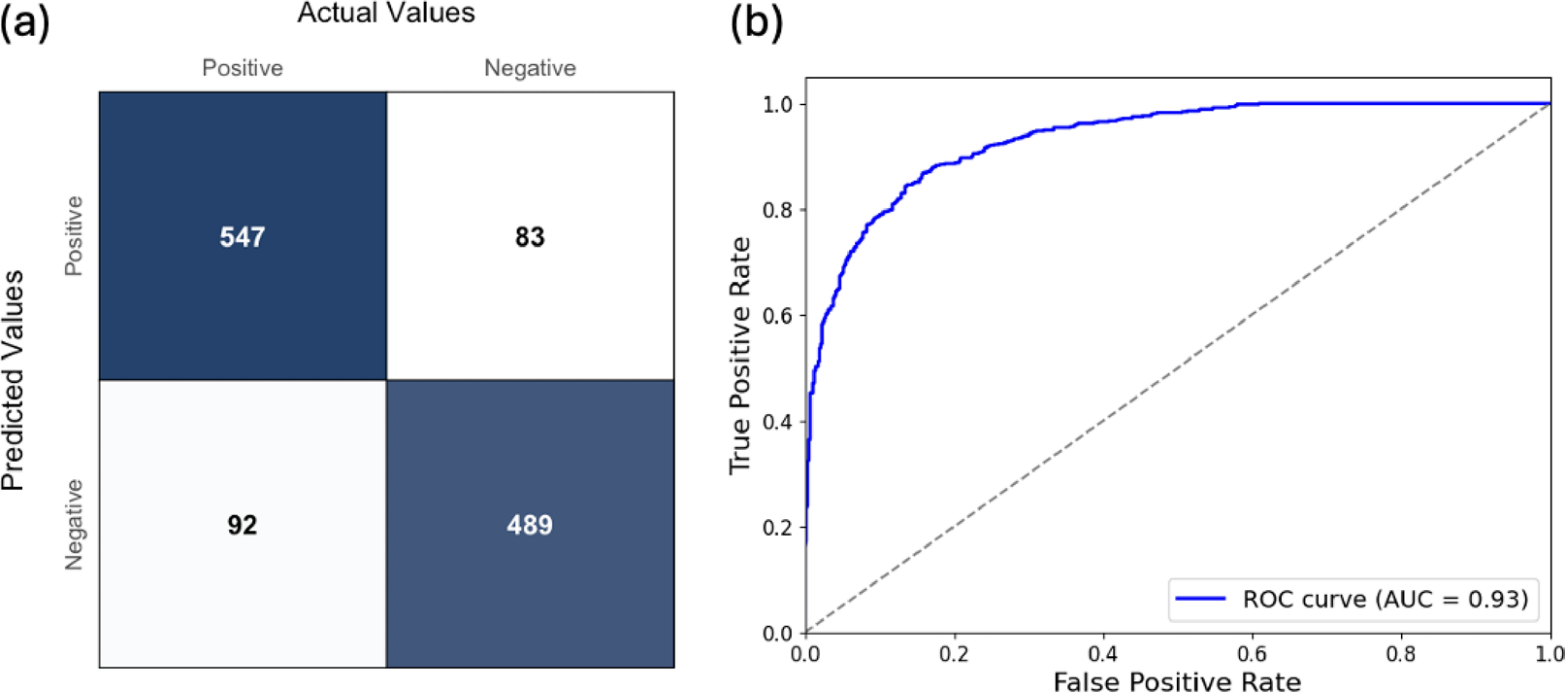
**(a)** Confusion matrix summarising the model’s detections for 30-second periods from the validation dataset. Numbers represent the count of frames correctly or incorrectly classified, predicted values shown on the y-axis and actual values on the x-axis. **(b)** receiver operating characteristic (ROC) curve showing the trade-off between true positive rate and false positive rate for the classification model. The Area Under the Curve (AUC) quantifies overall model performance, with values closer to 1 indicating better discrimination.

The final CNN achieved an overall accuracy of 85.6% in detecting fin whale 20 Hz pulses on the validation dataset. Precision and recall were 86.8% and 85.6%, respectively, resulting in an F1 score of 86.2%. The confusion matrix for the CNN’s output on the validation dataset (Fig. 3a) was produced with a threshold of 0.5 applied to the CNN’s output predicted probabilities. The receiver operating characteristic (ROC) curve, which computes true positive rates and false positive rates across thresholds from 0 to 1, had an area under the curve (AUC) of 0.93 (Fig. 3b). These results showed that the CNN could reliably detect fin whale pulses in the analysed acoustic dataset.

### Fin whale pulse detections

Acoustic recordings were analysed from two ocean-bottom seismometers (OBSs) and one ocean-bottom hydrophone (OBH). Across the entire survey period (JD156–218), the CNN detector identified the highest average hourly number of 30-second periods containing pulses (“positive frames”) on OBH56 (mean = 46.9, range = 1–119), followed closely by OBS18 (mean = 45.3, range = 1–116). OBS46 recorded the fewest detections, with an average of 36.1 positive frames per hour (range = 1–103). OBH56 and OBS18 had similar proportions of positive frames (39.1% and 37.7%, respectively), while OBS46 had a lower proportion (30.1%). Notably, these hourly trends were consistent across the survey, and preliminary analysis indicated no significant diel cycle in fin whale vocalisations, justifying our focus on hourly mean counts.

**Fig. 4:**
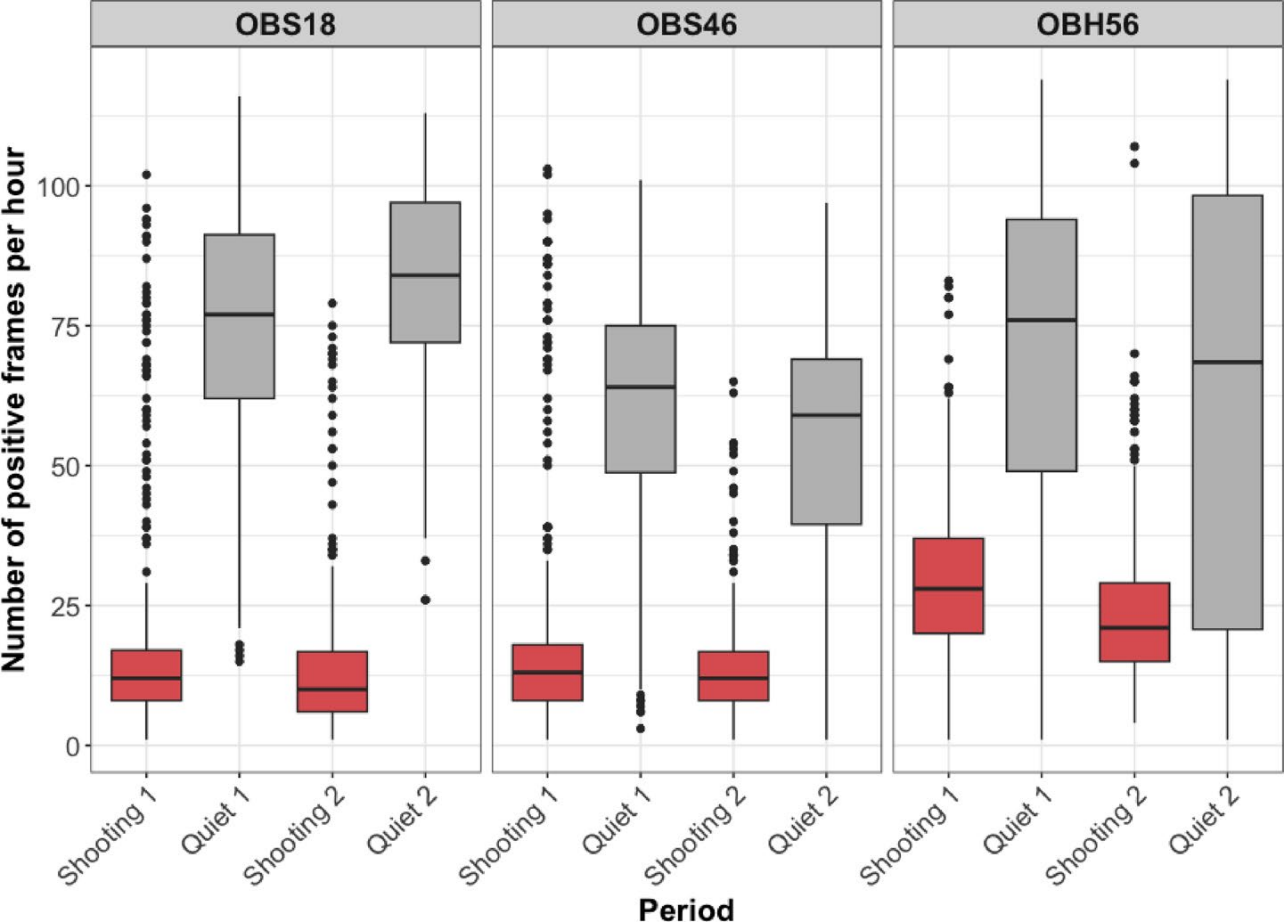
Positive frame counts per hour across different periods for each instrument. Each box represents the interquartile range, the horizontal line marks the median, whiskers extend to 1.5x the interquartile range, and the black circles represent outliers. Red boxes represent shooting periods, while grey boxes represent quiet periods.

**Fig. 5:**
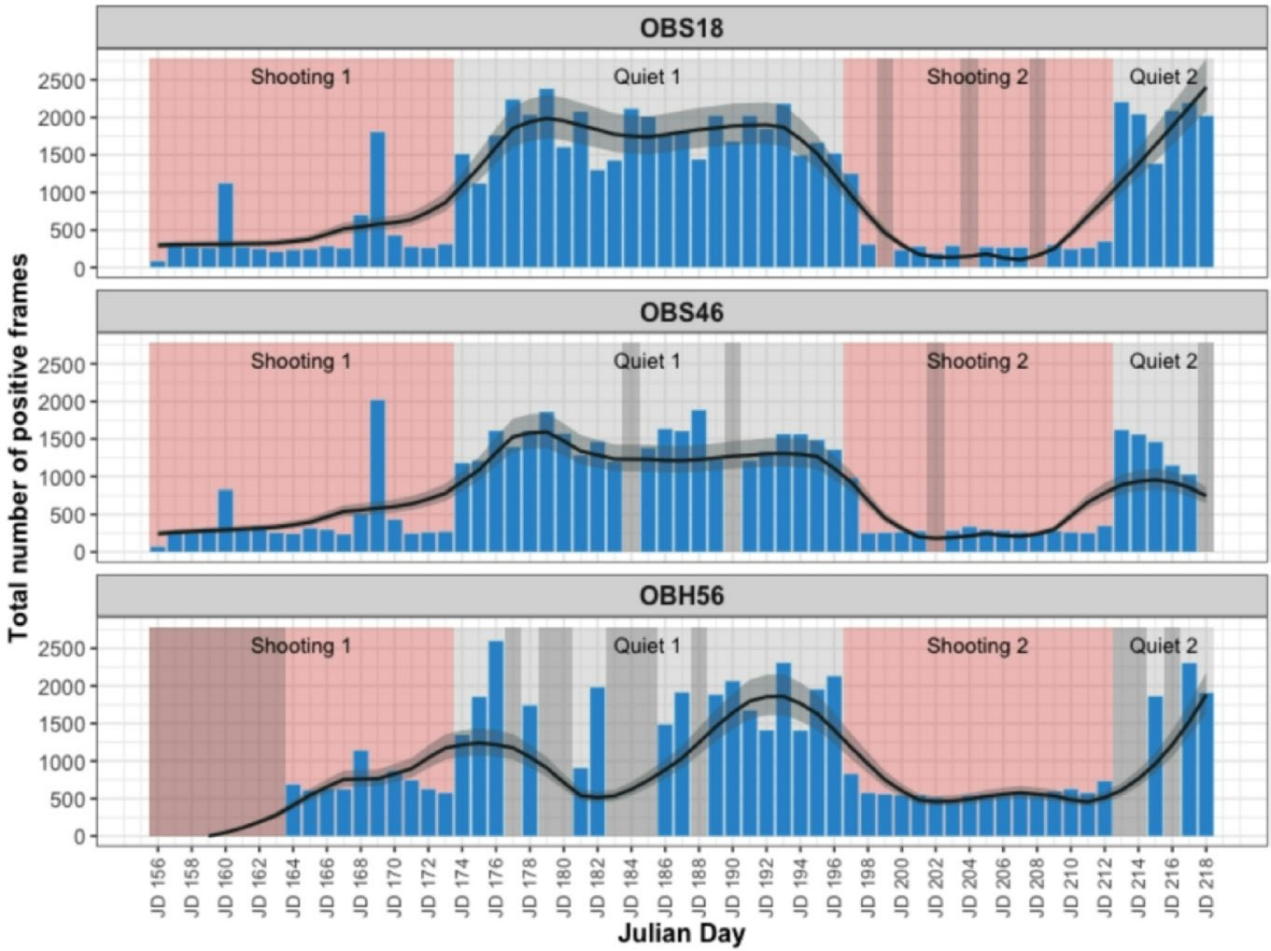
Daily total positive frame counts (bars) and Locally Estimated Scatterplot Smoothing (LOESS) smoothed trend lines for each instrument across Julian Days (JD). Shaded backgrounds indicate quiet and shooting (red) periods. The smoothed line was generated using a LOESS regression (span = 0.3) and is plotted with an error ribbon (semi-transparent band) that accounts for detection uncertainty, calculated by scaling the smoothed counts with instrument false positive (FPR = 0.158) and false negative (FNR = 0.132) rates. Days with corrupt data (grey vertical bands) were excluded from the analysis and treated as NAs.

Hourly counts showed clear temporal patterns across the four survey periods (Fig. 4) that we named as Shooting 1 (JD156–173, 18 days), Quiet 1 (JD 174–196, 23 days), Shooting 2 (JD 197–212, 16 days), and Quiet 2 (JD 213–218, 6 days). All instruments showed a sharp increase in detections at the onset of quiet periods, followed by a rapid decline when shooting resumed (Fig. 5). OBS18 detections were consistently higher during quiet periods, with Quiet 1 and Quiet 2 averaging 74.4 (range 15–116) and 83.0 (range 26–113) positive frames per hour, respectively. Counts declined during shooting periods, with Shooting 1 and Shooting 2 averaging 18.7 (range 1–102) and 14.0 (range 1–79) frames per hour. OBS46 exhibited a similar temporal pattern. Quiet periods yielded higher hourly counts (Quiet 1: 60.2, range 3–101; Quiet 2: 51.4, range 1–97) compared to shooting periods (Shooting 1: 18.4, range 1–103; Shooting 2: 13.7, range 1–65). For OBH56, the general pattern was similar, although counts during shooting periods were slightly higher than the other instruments (Shooting 1: 30.2, range 1–83; Shooting 2: 24.3, range 4–107), while quiet periods remained the periods of highest activity (Quiet 1: 69.9, range 1–119; Quiet 2: 60.2, range 1–119). Calling activity dropped markedly between quiet and shooting periods, with mean detections reduced by 78.2% on OBS18, 72.3% on OBS46, and 60.8% on OBH56 (Table 1).

Detection counts of fin whale calls varied strongly with survey period. A negative binomial mixed-effects model indicated strong reductions in detections during shooting relative to quiet periods, after accounting for instrument-level and day-level random effects (Supplementary Table S4a). Both shooting periods showed significant decreases in detections (Shooting 1: z = − 15.1 mean frames/h, *p* < 0.001; Shooting 2: z = − 17.1 mean frames/h, *p* < 0.001). Comparisons between quiet periods showed no significant overall change (z = − 1.07 mean frames/h, *p* = 0.29). Random effects revealed modest variation among instruments (SD = 0.12) and stronger day-specific variation within instruments (SD = 0.43), while the dispersion parameter (θ = 6.22) confirmed that the negative binomial distribution adequately accounted for overdispersion. Overall, detections were consistently higher during quiet periods than during shooting.

Model-derived Estimated Marginal Means (EMMs) supported these patterns: predicted hourly detections were highest during Quiet 1 (64.6 mean frames/h, 95% CI: 54.5–76.6) and Quiet 2 (57.1 mean frames/h, 95% CI: 44.7–72.8), and markedly lower during Shooting 1 (18.5 mean frames/h, 95% CI:15.4–22.2) and Shooting 2 (15.4 mean frames/h, 95% CI:12.8–18.5). Pairwise contrasts showed significant differences for all quiet–shooting comparisons (*p* < 0.001), while the contrasts between the two quiet periods and the two shooting periods were not significant. Full EMMs and pairwise comparisons of hourly detection counts are provided in Supplementary S4b and S4c.

**Table 1:** Mean hourly detections of fin whale pulse-positive frames per hour during quiet and shooting periods, with observed and worst-case (frame-based) masking corrections applied. Quiet means were averaged across Quiet 1 and Quiet 2; shooting means were averaged across Shooting 1 and Shooting 2. Corrected shooting means (positive frames h^−−1^) were calculated using the correction factor, assuming that every airgun shot overlapped a fin whale pulse. Percentage drops are relative to quiet means.

Instrument	Quiet mean (positive frames h^− 1^)	Shooting mean (frames h^− 1^)	Percentage drop (observed) (%)	Corrected shooting mean (frames h^− 1^, worst case)	Percentage drop (worst case) (%)
**OBS18**	76.2	16.6	78.2	23.2	69.6
**OBS46**	58.4	16.2	72.3	22.7	61.1
**OBH56**	67.8	26.6	60.8	37.2	45.1
**Mean**			**70.4**		**52.0**

### Maximum masking estimation

Application of the worst-case correction confirmed that seismic airgun activity had a strong suppressive effect on fin whale detections, even under the highly conservative assumption that every masked interval overlapped a fin whale call. Corrected mean hourly detection counts during shooting remained consistently lower than during quiet periods across all instruments (Table 1). The percentage decrease in calling activity when corrected for masking was 45.1–69.6%.

## Discussion

The results of this study demonstrated a clear association between seismic airgun activity and reduced fin whale vocalisation rates across the survey period. Across all three instruments, periods of active airgun shooting were characterised by marked declines in hourly detections of fin whale 20-Hz pulses compared to quiet periods. On average, vocal activity decreased by 70.4% when airguns were firing (Table 1), highlighting the scale of disruption caused by seismic survey noise. This decrease was supported by the mixed-effects model, which found statistically significant drops in detections during shooting relative to quiet periods (Supplementary S4a).

This observation contributes to a growing body of evidence that anthropogenic ocean noise has widespread and often adverse impacts on marine mammals^4,6,34^. However, the specific effects of seismic surveys on fin whales remain underexplored, largely due to the logistical challenges of conducting research in offshore environments and the complexity of possible behavioural responses to noise^14,35^. Interpretation of reduced call activity is further complicated by the context-dependent and still partially unresolved function of fin whale 20-Hz pulses^29,36^. While this uncertainty limits our ability to infer precise ecological consequences, it does not alter the clear behavioural changes in our data: fin whales reduce their vocal activity during periods of active shooting. The present study therefore fills an important gap by documenting repeated and prolonged reduction of fin whale calling behaviour during airgun use in the northeast Atlantic.

The temporal patterns observed suggested that the reduction in detections is not random but reflects behavioural change in response to seismic noise. Periods of quiet were marked by immediate increases in detections, often within one to two days of airgun cessation. During shooting, lower detections persisted throughout the periods, with Shooting 2 showing a rapid decline at onset (Fig. 5). Model-derived estimated marginal means showed the same pattern, with higher predicted detections during quiet periods than during either shooting period (Supplementary S4b and S4c). The elevated detections observed on JD160 and JD168–170 (Fig. 5) coincided with short operational pauses in airgun use reported in the cruise report, which occurred due to adverse weather conditions and equipment repairs^37^. While the underlying behavioural response cannot be determined, the temporal link indicates that these peaks were likely influenced by reduced noise exposure. The broader temporal patterns suggest that fin whales either reduced their calling rates, moved away from the survey area or exhibited a combination of these responses. While disentangling these mechanisms is beyond the scope of this study, both reduced vocal activity and displacement have been reported as common whale responses to seismic noise^14,20^.

In our dataset, fin whales maintained their responses across successive airgun shooting periods (Fig. 5), showing no signs of habituation, which would be characterised by diminished responses over time and continued calling during shooting^38^. We did not detect direct evidence for cumulative impacts but repeated responses could potentially contribute to such effects^14^. There is broader evidence that repeated or prolonged exposures to stressors, including noise, can be problematic for marine mammals, potentially leading to issues such as suppression of reproduction and accelerated aging^39^. Additionally, behavioural changes due to noise can lead to displacement from preferred habitats and negatively influence vital rates, as indicated by movement modelling^40^. If fin whales follow this broader pattern, and successive shooting periods are not fully isolated events, then repeated or prolonged exposure could exacerbate impacts on fin whale behaviour and habitat use.

These observed findings align with the outcomes of previous studies, albeit from a very limited literature base on fin whales. Altered song structure and reduced detections have been documented during seismic surveys, with vocal activity remaining supressed for two weeks after survey completion along with prompt decreases in fin whale pulses within the first 72 h of active shooting^14^. Additionally, the tracking of individual fin whales has shown movements away from survey vessels during the onset of airgun use^41^. At a broader taxonomic scale, declines in humpback whale singing have been associated with seismic survey noise, interpreted as both a reduction in calling behaviour and possible avoidance of noisy areas^20,21^. In contrast, a study using the same OBS dataset did not detect a clear correlation between seismic activity and fin whale vocal activity, likely due to the limited sample size and use of data predominantly within Quiet 1 period^23^. Overall, these studies have parallels with our results, strongly suggesting that the effects observed here are part of a wider pattern of seismic survey impacts on baleen whales.

Masking of fin whale pulses by seismic airgun activity represents an unavoidable limitation in this study. To assess its potential impact, we applied a conservative worst-case period-based correction, accounting for the number of airgun shots that could have obscured detections. Even under this extreme assumption, detections during shooting periods remained substantially lower than during quiet periods, with minimum guaranteed reductions of 45.1% (OBH56) and maximum of 69.6% (OBS18) (Table 1). These results indicate that observed decreases in fin whale vocal activity are not solely an artefact of masking. The estimates likely overstate the true effect of masking, as it is improbable that every airgun shot obscured a vocalisation. Incorporating this conservative analysis provides robustness to our findings and reinforces confidence that seismic activity reduces calling behaviour, rather than merely obscuring it.

The CNN demonstrated strong discrimination between fin whale pulses and background noise, including airgun pulses (Fig. 3). To account for potential limits in detection, we incorporated error margins from the CNN’s false positive and false negative rates into the detection counts and corresponding percentage reductions. Even with these adjustments and the worst-case masking correction, reductions in detected pulses remained substantial, thus, reinforcing that the observed declines reflect genuine behavioural responses rather than artefacts of detection or masking limitations (also see Supplementary Fig. S5).

A primary limitation of this study is the absence of baseline data prior to the initiation of airgun shooting. Without pre-shooting measurements, it is not possible to determine the “normal” vocalisation activity or natural variability of the fin whales in the absence of anthropogenic disturbance. Given the clear impact of seismic surveys on fin whale vocalisations observed here, future survey designs could incorporate a secondary aim of assessing these effects. Deploying passive acoustic recorders, perhaps from a different vessel, for a period before the start of shooting would provide a baseline of typical calling behaviour, allowing a more robust assessment of how survey activity alters vocal activity. Additional limitations arise from the spatial configuration of the array and the vessel track. While multiple instruments were deployed across the survey area, behavioural responses may have varied spatially depending on proximity to the airgun source as well as habitat features^19,22^. Future work could expand analysis across all of the instruments recovered with useful data to enable quantification of response variability across the population. Finally, combining visual or tagging data alongside acoustic monitoring could help distinguish between reduced vocalisation versus actual displacement from the area^19,42^.

The consistent and substantial reduction in pulse-positive frames across all instruments, averaging 70.4% during active airgun shooting and 52.0% when accounting for a worst-case masking scenario, underscores the significant influence of anthropogenic noise on fin whale vocalising behaviour. These results suggest that seismic survey activity can induce an immediate response and may create progressive behavioural responses, likely reflecting a combination of decreased calling and temporary avoidance of noisy areas. Given the widespread use of seismic surveys and their capacity to propagate sound over long distances^11–13^, these findings raise concerns about potential long-term impacts on fin whale communication, energy expenditure, and habitat use^38^. From a conservation and management perspective, these results highlight the necessity of considering the timing, intensity, and spatial coverage of seismic surveys in areas important for baleen whales, especially given that the reach of airgun noise extends far beyond the immediate survey zone^12^. Mitigation measures, such as exclusion zones, or seasonal restrictions, may help reduce behavioural disruptions^18,22^. Alternative source technologies could also provide a quieter approach to seismic exploration. For example, marine vibroseis systems use a vibrating plate or shell to generate longer-duration, lower-amplitude signals that are variable in frequency to deliver the acoustic energy required for seismic imaging, and these are considered a less environmentally disruptive alternative to the impulsive signals of conventional airgun sources^43^. More broadly, this study highlights the importance of integrating acoustic monitoring into activities that produce anthropogenic noise to better understand the impacts of permitted sound-generating operations on marine mammals and inform responsible management of such activities.

## Methods

### OBH and OBS acoustic data

The acoustic data used in this study were collected during the Galicia3D seismic survey in 2013 and are openly available through PANGAEA^44^. A total of 72 ocean-bottom instruments were deployed to continuously record the ocean’s soundscape throughout the survey period, comprising of systems from the British Ocean Bottom Instrumentation Consortium (OBIC)^45^ and GEOMAR^46^.

To assess the impact of the survey on whale vocalisations, it was not necessary to analyse the whole dataset, which would have introduced significant redundancy since the same sets of vocalisations were detected on multiple instruments^23^. We analysed data from two ocean-bottom seismometer (OBS) instruments (OBS18 and OBS46) that recorded at 250 Hz sample rate and one ocean-bottom hydrophone (OBH) instrument (OBH56) that recorded at 200 Hz (Fig. 2), selected to provide both broad spatial coverage and variation in recording systems for training and evaluating the CNN. These instruments had a spacing of 30–40 km and are likely to have recorded independent sets of vocalisations. Data were taken from the hydrophones of OBS18 and OBH56 and the vertical geophone of OBS46. Further details of these recording instruments are outlined in supplementary material (Supplementary Table S1).

The data analysed for this study spanned from 5th June (Julian Day (JD)156) to 6th August (JD 218). This timeframe included two periods of active seismic shooting operations (Shooting 1: JD156-173; Shooting 2: JD 197–212) and two quiet periods with no airgun activity (Quiet 1: JD 174–196 during vessel repairs; Quiet 2: JD 213–218 after survey completion). For the purposes of analysis, any day during which airgun shooting occurred at any time was classified as a shooting day. These four periods provided the framework for evaluating fin whale acoustic activity in relation to shooting operations.

### Manually labelled training dataset

To train and assess the performance of the CNN algorithm for its automated detection of the fin whale pulses, a manually labelled subset of data served as training and validation datasets. This manual signal labelling process was carried out in HydroSeek, a custom signal labelling application for passive acoustic monitoring purposes^47^. This software converted the acoustic data into 30-second spectrogram frames allowing the user to manually label signals present in the time-frequency domain. Each processed frame was assigned a binary score, corresponding to the presence or absence of signals. Within HydroSeek, label classifications were made using three spectrogram views of varying input parameters for multi-scale analysis of the acoustic signals present. The three spectrogram configurations were: FFT 256 points, 78% overlap, 1–99 Hz; FFT 256 points, 86% overlap, 1–80 Hz; and FFT 300 points, 83% overlap, 18–30 Hz. The dynamic range parameter was set between − 30 and 10 dB, but due to differences in sample rates, these dynamic range values required occasional minor adjustment for the clearest spectrograms.

To minimise subjectivity and maintain consistency in the manual labelling process, clear criteria were established for each label classification (Supplementary Table S2). Despite these criteria, some subjectivity is unavoidable^48^. To maintain consistency, the labelling process was limited to two trained analysts. A shared blind review of 10% of the dataset indicated a 3.75% inter-analyst disagreement rate. In all cases, the annotation of the original labelling was retained for analyses.

During this process, 30 min of acoustic data were manually labelled in 2-hour intervals for the first complete day of the Shooting 1 (JD156), Quiet 1 (JD175) and Shooting 2 (JD198) periods from OBS18, OBS46 and OBH56. This range of instruments and days ensured the dataset contained a comprehensive repertoire of the signals present (Table S3).

### Machine learning

#### Model structure

To detect fin whale 20 Hz calls, we used a convolutional neural network (CNN) classifier by adapting an established image-recognition architecture and training it on spectrogram representations of the acoustic data. We implemented the CNN using the EfficientNet^49^ model family’s baseline architecture, EfficientNetB0, pre-trained on the ImageNet dataset^50^. Global average pooling^51^ was applied to the output of the EfficientNetB0 feature extractor before feeding it into the classification part. This classification part consists of two fully connected layers with 512 and 256 neurons, each with a rectified linear unit activation function and followed by dropout layers^52^ with the rates of 0.5 and 0.2, respectively. The final output layer uses a softmax activation with two units for binary classification (i.e., presence vs. absence of fin whale calls). The dense layers in the classification part were initialized with Glorot uniform^53^ for the weights and zeros for the bias vectors and the EfficientNetB0 feature extractor with the pre-trained parameters. The network was trained with full fine-tuning, meaning that the entire network was trained on the target dataset, after parameter initialisation. The same network architecture has been employed in several underwater acoustics classification studies^54–56^.

#### Input representation

Training inputs consisted of 30 s segments, downsampled to 200 Hz and transformed into spectrograms using FFT 256 points and 75% overlap with frequency band-limiting applied between 4 and 80 Hz. Per channel energy normalisation (PCEN) was used to enhance signal detection under noisy conditions by suppressing background energy and emphasising transient low-frequency calls^57^. PCEN hyperparameters were set following previous work on tuning them empirically, which demonstrated their efficacy compared with alternative input representations^54^. To avoid bias from temporal sequence, each spectrogram was treated independently of its chronological context. The final input dimensions were 97 time frames by 90 frequency bins with red-green-blue conversion applied, 97 × 90 × 3.

#### Training dataset

The model was trained using manually labelled data from OBS18, OBS46, and OBH56 across JDs 156, 175, and 198, covering both survey and quiet periods. Positive samples contained ≥ 1 fin whale pulses, while negative samples were drawn from a range of acoustic backgrounds, including airgun pulses and ambient noise. To minimise class imbalance, approximately equal numbers of positive and negative frames were used for training (3,099 positive, 2,892 negative frames), totalling approximately 50 h of labelled data. A full breakdown of the training datasets is provided in the supplementary material (Supplementary Table S3).

#### Model training

Training was conducted by using a categorical cross-entropy loss function. We use an adaptive moment estimation optimizer with exponential learning rate decay^58^. The exponential decay schedule used an initial rate of 0.001 and a decay rate of 0.75 at every 90 steps with a staircase function.

#### Validation

An unseen dataset of 30 s spectrogram samples containing a similar number of positive (*n* = 639) and negative (*n* = 572) frames was used for validation (Supplementary Table S3). Model performance was quantified using multiple metrics. Overall accuracy provided a baseline measure of correct classifications. Precision was defined as the proportion of CNN detections that were true positives, whereas recall represented the proportion of true fin whale pulses correctly detected by the model. The F1 score, the harmonic mean of precision and recall, was included as a summary metric that balances the trade-off between false positives and false negatives. In addition, Receiver Operating Characteristic (ROC) analysis was performed to evaluate model performance across all decision thresholds, with the Area Under the Curve (AUC) used to summarise the model’s overall discriminative ability independent of the choice of threshold.

#### Large-scale application

The final CNN was applied to the full acoustic dataset from JD 156–218, spanning 63 days of continuous recordings across all three instruments. Each 30 s segment was independently classified, producing > 180,000 outputs per instrument. A probability threshold of 0.5 was used to convert model outputs into binary classifications. This optimal ROC-derived threshold value was 0.51, and we therefore used the rounded value of 0.5, consistent with standard practice in binary classification^59^. The same threshold was used for evaluating the model and for generating daily detection counts. Processing was carried out on Google Colab using a T4 High-RAM runtime. In total 25 days contained corrupt data, likely due to data conversion or transcription issues. These data were excluded from further analysis.

### Statistical analysis

Detection count data generated by the CNN were analysed to evaluate fin whale acoustic activity in relation to seismic shooting operations. Each positive ‘detection’ corresponded to a 30-second frame containing at least one fin whale pulse. Daily detection counts were summarised separately for each instrument and partitioned into the four survey periods to observe phase changes.

CNN-derived detections were first aggregated into hourly means to reduce fine-scale variability and to standardise comparisons across the full survey period. Periods with corrupted data files were treated as missing (NA) and excluded from all analyses. To assess differences in hourly mean detection rates among survey periods, we fitted a negative binomial Generalised Linear Mixed-effect Model (GLMM) using the glmmTMB package in R^60^. The model specified Period as a fixed effect and incorporated two random-effect terms: (1| Instrument) to account for variation among recorders, and (1| Instrument: JD) to account for day-level temporal autocorrelation within each instrument. A negative binomial error structure was selected after the initial Poisson models showed substantial overdispersion. Model assumptions were evaluated using the DHARMa package^61^, including dispersion tests, zero-inflation tests and simulation-based residual diagnostics.

Post hoc comparisons between survey periods were conducted using Estimated Marginal Means (EMMs) using the emmeans package in R^62^. Pairwise contrasts were adjusted using Tukey correction, and estimates were reported on the response scale (mean detections per hour) with 95% confidence intervals. This approach allowed direct comparison of call occurrence rates between active shooting and quiet conditions for each instrument and location independently. Model residuals were visually inspected to confirm distributional assumptions and goodness of fit. All analyses were conducted in R (v4.4.1)^63^.

### Maximum masking estimation

To assess the inherent limitation presented by the masking of fin whale pulses by airgun shot signals within the data, we estimated the fraction of spectrogram frames likely to be obscured during active shooting. Airgun pulses occurred on average every 16 s, with each pulse producing a masking duration of 5.5 s (Mean = 5.50, range = 2.73–7.73 s). Pulse characteristics were obtained through manual review of 20 representative pulses sampled across both survey legs (Fig. 1b).

We used the recorded number of airgun shots per shooting period to estimate actual masked time. For each period we multiplied the shot count by the mean masking duration (5.5 s) to obtain total masked seconds and then converted to hours. For Shooting 1 (*n* = 66,898 shots), this produced 102 h masked out of 432 h of surveying. For Shooting 2 (*n* = 84,417 shots), this produced 129 h masked out of 384 h.

To incorporate the effect of this masked time into the detection analysis, we derived a single average masking correction factor across both shooting periods. The proportion of shooting time that was masked was calculated (23.5% in Shooting 1; 33.6% in Shooting 2), producing an overall mean masked fraction of 28.7%. Assuming detections are equally likely to occur within masked and unmasked intervals, the expected reduction in detectability is proportional to the masked time. Therefore, we applied a correction factor, calculated as 1/ (1-0.287) = 1.40, to the observed mean hourly detections during shooting to account for calls potentially lost due to masking. This approach allowed us to assess the robustness of observed decreases in calling during active airgun activity.

## Supplementary Information

Below is the link to the electronic supplementary material.

Supplementary Material 1

## Data Availability

The acoustic data used in this study were collected during the Galicia3D seismic survey in 2013 and are openly available via PANGAEA (Bayrakci et al., 2022): [https://doi.org/10.1594/PANGAEA.940656]. All code used for data processing, statistical analysis, and the CNN model is available from the corresponding author upon reasonable request.
