# Supplementary material for: Seismic survey noise reduces fin whale vocalisations offshore northwestern Spain
Source: Sci Rep. 2026 Feb 25;16:10449. doi: 10.1038/s41598-026-40903-x (PMC13031941; doi:10.1038/s41598-026-40903-x)

## **Supplementary Information**

Table S1 Specifications of the ocean-bottom recording instruments OBS18, OBS46 and OBH56 used for this study. (Bayrakci et al., 2022; Papenberg and Klaeschen, 2014).

|  | Instrument | | |
| --- | --- | --- | --- |
| System | **OBS18** | **OBS46** | **OBH56** |
| System | Ocean Bottom Instrumentation Consortium (OBIC) | | GEOMAR |
| Number of recording channels | 4 | | |
| Structure | 3-component gimballed geophone package and single broadband hydrophone recording at 4.5 Hz | | 3-component seismometer and a single 4. 5 Hz natural frequency hydrophone |
| Recording channel used | Broadband Hydrophone (CH3) | Vertical geophone (CH0) | Hydrophone |
| Sampling frequency (Hz) | 250 | | 200 |
| Recording period available | 05/06/2013 to 12/08/2013 (JD 156-224) | 05/06/2013 to 13/08/2013 (JD 156-225) | 05/06/2013 to 09/09/2013 (JD 156-252) |

*Table S2 The names and criteria of each label applied during the manual labelling process of the sample data from JD 156, 175 and 198 over OBS18, OBS46 and OBH56. Any frame annotated as any of the following call labels was classed as vocalisation positive.*

| **Label** | **Criteria** |
| --- | --- |
| **‘Fin whale calls’** | Multiple strong fin whale vocalisation pulses:   - Clear classic down-sweeping shape - Strong signal (green-yellow coloured in spectrograms A, B and C) - Typically, ranging from ~18-28 Hz - ~1 second duration per call - Typical call train Inter-Note Intervals (INI) of 5-20 seconds - Typically visible in the Context Plot as well as the sub-spectrograms |
| **‘1 call’** | A singular call with acoustic characteristics that match the criteria in ‘Fin whale calls’ |
| **‘Faint calls’** | Multiple faint fin whale vocalisations of similar characteristics as ‘Fin whale calls’ but:   - Lower in signal strength (light blue-green coloured in Spectrograms A, B and C) - Can be less broad in vertical frequency range, as narrow as ~4 Hz - Can have a less pronounced down-sweeping shape - Typically shorter individual pulse duration (<1 second) - More variable INIs, usually ranging from ~1-20 seconds. |
| **‘1 faint call’** | A singular call with acoustic characteristics that match the criteria in ‘Faint calls’ |
| **‘Shooting’** | Frames in which active seismic surveying is occurring, evident by airgun shot signals within the frame. These signals are:   - Strong broadband signals, usually spanning the entire spectrogram frequency axis but can be shorter in frequency range based on shooting proximity. - Typically occur in sequences of four signals: the first signal being the actual shot signal with the strongest return and the remaining three reflection signals of the initial shot progressively decreasing in frequency range and strength - Typical initial shot intervals ranged from ~32-16 seconds depending on the survey period. |
| **‘Noise’** | Noise present anywhere within the frame, typically occurring as horizontal bands or patches contrasting the ambient noise. |

*Table S3 Sample sizes and label composition of positive and negative training and validation datasets used for the convolutional neural network (CNN) detection model. Note: unclassified frames are those that were not annotated to contain any of the signals labelled.*

| Input dataset | Classification | Sample size | Corresponding labels |
| --- | --- | --- | --- |
| Training | Positive | 2,460 | - ‘Fin whale call’/ ‘1 call’ - ‘Faint calls’/ ‘1 faint call’ |
|  | Negative | 2,320 | - Unclassified frames - ‘Shooting’ - ‘Noise’ |
| Validation | Positive | 639 | - Fin whale call’/ ‘1 call’ - ‘Faint calls’/ ‘1 faint call’ |
|  | Negative | 572 | - Unclassified frames - ‘Shooting’ - ‘Noise’ |
| Total of 3,099 positive frames and 2,892 negative frames input into the CNN model | | | |

*Supplementary S4*

*Table S4a Negative binomial GLMM summary for detection counts.*

**Model:** Count ~ Period + (1 | Instrument) + (1 | Instrument:JD)

| Parameter | Estimate | Std. Error | z value | p-value |
| --- | --- | --- | --- | --- |
| (Intercept) | 4.169 | 0.087 | 48.09 | **<0.0001** |
| Period Quiet 2 | -0.125 | 0.117 | -1.07 | 0.286 |
| Period Shooting 1 | -1.252 | 0.083 | -15.13 | **<0.0001** |
| Period Shooting 2 | -1.436 | 0.084 | -17.09 | **<0.0001** |

**Random effects:**

| Group | Variance | SD |
| --- | --- | --- |
| Instrument | 0.014 | 0.118 |
| Instrument:JD | 0.187 | 0.433 |

Model diagnostics:

- Number of observations: 4151
- Dispersion parameter (θ): 6.22
- DHARMa dispersion test: 0.436, p < 0.0001
- Zero-inflation test: ratioObsSim = 0, p = 0.176

*Table S4b Estimated marginal means (EMMs) of hourly detection counts per period.*

| Period | EMM (frames/hr) | SE | 95% CI |
| --- | --- | --- | --- |
| Quiet 1 | 64.6 | 5.60 | 54.5 – 76.6 |
| Quiet 2 | 57.1 | 7.11 | 44.7 – 72.8 |
| Shooting 1 | 18.5 | 1.72 | 15.4 – 22.2 |
| Shooting 2 | 15.4 | 1.45 | 12.8 – 18.5 |

Back-transformed from the log scale; SE = standard error; 95% CI = 95% confidence interval.

### *Table S4c Pairwise comparisons of EMMs (Tukey-adjusted)*

| Contrast | Estimate (log scale) | SE | z ratio | p-value |
| --- | --- | --- | --- | --- |
| Quiet 1 – Quiet 2 | 0.125 | 0.117 | 1.068 | 0.7093 |
| Quiet 1 – Shooting 1 | 1.252 | 0.083 | 15.133 | **<0.0001** |
| Quiet 1 – Shooting 2 | 1.436 | 0.084 | 17.088 | **<0.0001** |
| Quiet 2 – Shooting 1 | 1.127 | 0.122 | 9.249 | **<0.0001** |
| Quiet 2 – Shooting 2 | 1.311 | 0.123 | 10.681 | **<0.0001** |
| Shooting 1 – Shooting 2 | 0.184 | 0.091 | 2.025 | 0.1786 |

*Supplementary S5*

### *Figure S5 Confusion matrices on the validation dataset for quiet (a) and shooting (b) periods. The F1 scores are 0.89 for the quiet period and 0.85 for the shooting period, demonstrating that despite class imbalance, the model maintains balanced performance with no skewness to the number of false positives or false negatives.*

b)

a)


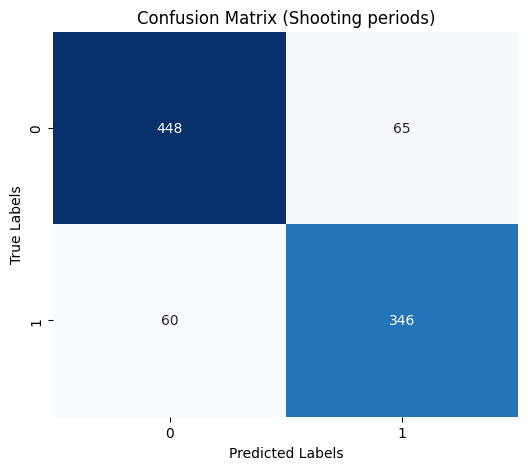

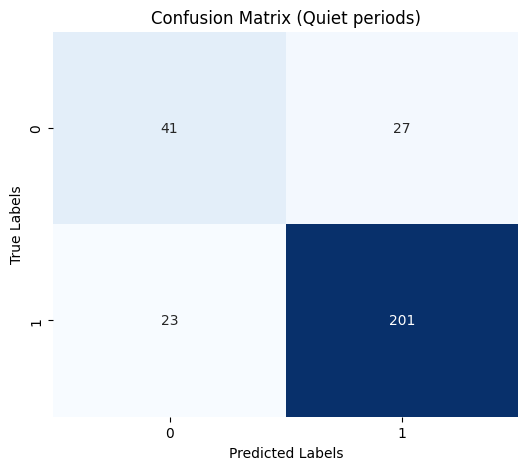

Supplement: Supplementary file 1 — Supplementary Material 1 [file 41598_2026_40903_MOESM1_ESM.docx]
